# Left pulmonary artery in 22q11.2 deletion syndrome. Echocardiographic evaluation in patients without cardiac defects and role of *Tbx1* in mice

**DOI:** 10.1371/journal.pone.0211170

**Published:** 2019-04-01

**Authors:** Gioia Mastromoro, Giulio Calcagni, Paolo Versacci, Carolina Putotto, Marcello Chinali, Caterina Lambiase, Marta Unolt, Elena Pelliccione, Silvia Anaclerio, Cinzia Caprio, Sara Cioffi, Marchesa Bilio, Anwar Baban, Fabrizio Drago, Maria Cristina Digilio, Bruno Marino, Antonio Baldini

**Affiliations:** 1 Department of Pediatrics, Sapienza University of Rome, Rome, Italy; 2 Department of Pediatric Cardiology and Cardiac Surgery, Bambino Gesù Pediatric Hospital and Research Institute, Rome, Italy; 3 Institute of Genetics and Biophysics “Adriano Buzzati Traverso”, CNR, Naples, Italy; 4 Department of Molecular Medicine and Medical Biotechnologies, University of Naples Federico II, Naples, Italy; Scuola Superiore Sant'Anna, ITALY

## Abstract

**Introduction and hypothesis:**

Patients with 22q11 deletion syndrome (22q11.2DS) present, in about 75% of cases, typical patterns of cardiac defects, with a particular involvement on the ventricular outflow tract and great arteries. However, in this genetic condition the dimensions of the pulmonary arteries (PAs) never were specifically evaluated.

We measured both PAs diameter in patients with 22q11.2DS without cardiac defects, comparing these data to a normal control group. Moreover, we measured the PAs diameter in *Tbx1* mutant mice. Finally, a cell fate mapping in *Tbx1* mutants was used to study the expression of this gene in the morphogenesis of PAs.

**Methods:**

We evaluated 58 patients with 22q11.2DS without cardiac defects. The control group consisted of 54 healthy subjects, matched for age and sex. All cases underwent a complete transthoracic echocardiography. Moreover, we crossed *Tbx1*^*+/-*^ mice and harvested fetuses. We examined the cardiovascular phenotype of 8 wild type (WT), 37 heterozygous *(Tbx1*^*+/-*^*)* and 6 null fetuses *(Tbx1*^*-/-*^*)*. Finally, we crossed *Tbx1*^*Cre/+*^mice with *R26R*^*mT-mG*^ Cre reporter mice to study *Tbx1* expression in the pulmonary arteries.

**Results:**

The echocardiographic study showed that the mean of the LPA/RPA ratio in 22q11.2DS was smaller (0.80 ± 0.12) than in controls (0.97 ± 0.08; p < 0.0001).

Mouse studies resulted in similar data as the size of LPA and RPA was not significantly different in WT embryos, but in *Tbx1*^+/-^ and *Tbx1*^-/-^ embryos the LPA was significantly smaller than the RPA in both mutants (P = 0.0016 and 0.0043, respectively). We found that *Tbx1* is expressed near the origin of the PAs and in their adventitia.

**Conclusions:**

Children with 22q11.2DS without cardiac defects show smaller LPA compared with healthy subjects. Mouse studies suggest that this anomaly is due to haploinsufficiency of *Tbx1*. These data may be useful in the clinical management of children with 22q11.2DS and should guide further experimental studies as to the mechanisms underlying PAs development.

## Introduction

Patients with 22q11.2 deletion syndrome (22q11.2DS) present specific conotruncal defects [1,2, 3] including tetralogy of Fallot with or without pulmonary atresia [4, 5], Truncus Arteriosus [6, 7], Interrupted Aortic Arch [8, 9], other aortic arch anomalies or minor congenital heart defects [10, 11], and ventricular septal defect [12].

In more than 90% of them a 3 Mb deletion was detected [13], spanning LCR22-A to LCR22-D that contains at least 30 genes including *TBX1* in the proximal region. This encodes a T-box transcription factor identified as the major player of this syndrome throughout both modeling mice [14–17] and mutational analysis in patients [18]. In *Tbx1* mutant mice some cardiovascular anomalies similar to those found in 22q11.2DS patients have been described [15, 19]. These observations can be explained by the fact that *Tbx1* is expressed in precursors of outflow tract cells and its loss of function reduces cell contribution to the outflow tract [20–22].

Additional anomalies of pulmonary arteries (PAs) including diffuse hypoplasia, discontinuity, and crossing, were sporadically reported in 22q11.2DS patients [1, 3, 23–26] but not extensively studied. The aim of this study is to investigate the dimensions of both PAs in patients with this syndrome. In addition, we have analyzed the PAs diameters in *Tbx1* knockout mice and found that its haploinsufficiency is associated with PAs asymmetry, indicating that this gene is the candidate for the PA phenotype reported here.

## Materials and methods

This is a prospective multicentric observational and experimental study conducted in three different Italian Centers: Department of Pediatrics, Sapienza University of Rome, Bambino Gesù Children’s Hospital and Research Institute, and Institute of Genetics and Biophysics of National Research Council, Naples.

Mouse studies were carried out at the Institute of Genetics and Biophysics under the auspices of the animal protocol 257/2015-PR (licensed to the AB lab) reviewed, according to Italian regulations, by the Italian Istituto Superiore di Sanità and approved by the Italian Ministero della Salute. The laboratory applies the "3Rs" principles to minimize the use of animals and to limit or eliminate suffering.

### Echocardiographic study

Patients data were collected from our hospital database of patients attending the Pediatric Cardiology Division of Sapienza University and Bambino Gesù Children Hospital from October 2010 to April 2017. Informed consent was obtained from each patient (or legal guardians). The echocardiographic measurements are derived from routine ultrasound exams. The study conforms to the ethical guidelines of the 1975 Declaration of Helsinki.

We included 58 pediatric and adult patients with 22q11.2DS without intracardiac malformations. Six of them presented isolated non-obstructive abnormalities of aortic arch or epiaortic vessels. We excluded from our cohort subjects with cardiac defects because of possible flow-related bias in our PAs measurements. The group of 58 cases consisted of 23 females (39.6%) and 35 males (60.4%), mean age of 12.8 ±10 years, with a mean body surface area (BSA) of 1.17±0.5; our control group consisted of 54 subjects (41% females) with a mean age of 10.8 ±10 years and a mean BSA of 1.18±0.5.

All patients underwent genetic counseling, and fluorescent in situ hybridization was performed to confirm the specific microdeletion. Echocardiographic measurements were compared with healthy subjects matched for age, sex, and BSA. All patients and healthy controls underwent a complete transthoracic echocardiographic examinations using GE Vivid E9 (Medical Systems, Oslo, Norway) with M6SD and 7S convex probe and Philips Ie33 Machine (Philips Medical Systems, Andover, MA) with X-5 and X-7 probes. M Mode, 2-Dimensional, and Doppler examinations were performed in all subjects. In particular, pulmonary branches were measured in parasternal short axis view during systole. Aortic arch anomalies were diagnosed in jugular view. According to BSA, Z score values were reported for M Mode results and for 2D PA branches diameters. Images were digitally stored and measurement were made offline according to the American Society of Echocardiography guidelines by two independent readers for both centers (GM, PV, and GC, EP).

### Mouse studies

To perform phenotypic analyses, *Tbx1*^*+/-*^ mice [15] were intercrossed and pregnant females (3–6 months-old) were sacrificed using CO_2_ inhalation at plug day (E) 18.5, and fetuses harvested. Prior to observation. fetuses were washed in PBS and dissected under a Zeiss Stemi 2000-CS Stereo Microscope. Photographs were taken using a Z-stack software. In order to improve the view, we injected ink into the pulmonary trunk. Overall we have dissected and examined the cardiovascular phenotype of 8 wild type (WT), 37 heterozygous (*Tbx1*^+/-^), and 6 null fetuses (*Tbx1*^-/-^). PA measurements (in pixels) were taken from good quality images at the same magnification using software tools. Statistical significance was evaluated using the Mann-Withney test. To reveal *Tbx1*-expressing cells and their descendants, we crossed *Tbx1*^*Cre*/+^ mice [27] with *Rosa*^*mT*-*mG*^ mice a Cre reporter [28]. Hearts of E18.5 *Tbx1*^*Cre*/+^; *Rosa*^*mT*-*mG*^ embryos were dissected, photographed whole mount under Stemi 2000-CS Stereo Microscope with epifluorescence illumination, and then processed for cryosectioning. Sections were immunostained with an anti PECAM1 antibody (mouse monoclonal 2H8, Thermo Fisher MA3105, diluted 1:200) and/or an anti GFP antibody (Abcam ab13970, 1:800) as described elsewhere [21]. Sections were photographed using a Leica fluorescence microscope. Digital images were mounted using Photoshop to generate the figures shown here.

## Results

### 22q11.2DS patients have smaller LPAs than controls, independently from intracardiac anomalies

We identified 58 patients with isolated abnormalities of aortic arch or epiaortic vessels disease according to our criteria. Table 1 summarizes major clinical findings. All 22q11.2DS patients had variable expressivity and incomplete penetrance of dysmorphic features typical of the syndrome. A similar group of healthy volunteers was analyzed (Table 2). Using echocardiography, we have measured the diameter of PAs in a healthy subgroup and we found that LPAs were smaller than RPAs (9.4± 2.4 vs 10.0±2.8. P < 0.05). This finding was confirmed also in in 22q11.2DS cases, which exhibited LPAs measurements smaller than RPAs (8.2± 2.4 vs 9.4±2.4; P = 0.016) and in Z score (-1.57± 1.2 vs -0.65± 1.0; P<0.001). No differences were found comparing diameters of RPAs in cases and controls. In contrast, LPA/RPA ratios showed a significant difference between the two groups: 0.80±0.09 in cases vs 0.95±0.11 in controls (P < 0.001) (Tables 1 and 2).

**Table 1:** Echocardiographic data of 22q11.2DS patients without intracardiac defects.

Case	Sex	Ao Arch Anomalies	PAs Anomalies	Age	Weight (kg)	High (cm)	BSA	LPA d	z-score LPA	RPA d	z-score RPA	LPA/RPA
**1**	M		CPAs	0	8	79	0.42	3.9	-2.95	4.1	-3.43	0.95
**2**	M			1	8.7	80	0.44	4.7	-2.03	5.7	-1.63	0.82
**3**	F			1	10	72	0.46	7.7	0.8	8	0.29	0.96
**4**	F			1	9.7	75	0.46	5.4	-1.32	6.8	-0.7	0.79
**5**	F	RAA		2	12	83	0.53	7.8	0.29	9.3	0.56	0.84
**6**	M			2	8.5	80	0.44	5.4	-1.15	6.6	-0.69	0.82
**7**	M			2	13	86	0.56	6.6	-0.93	7.9	-0.67	0.84
**8**	M		CPAs	2	11	83	0.51	5	-2.19	10	1.2	0.50
**9**	F	RAA, Retroesophageal ARSA		3	14	93	0.6	6.1	-1.67	6.8	-1.87	0.90
**10**	F			3	13	95	0.59	5.4	-2.28	6.3	-2.21	0.86
**11**	M			4	17	100.3	0.69	9.1	0.24	10	-0.04	0.91
**12**	M			4	28	107	0.93	5.7	-3.47	7.8	-2.47	0.73
**13**	F			4	16	100	0.67	5.6	-2.55	6.5	-2.53	0.86
**14**	F			4	12.4	93	0.57	5.4	-2.15	6.5	-1.88	0.83
**15**	M	DAA		4	15	102	0.65	6.6	-1.47	8.3	-0.94	0.80
**16**	M			4	17	106	0.71	5.4	-2.96	8.4	-1.18	0.64
**17**	M			4	18	101	0.72	7.3	-1.2	9.2	-0.67	0.79
**18**	F	Retroesophageal ARSA		5	15.5	105	0.67	5.8	-2.38	6.9	-2.18	0.84
**19**	M	RAA, Retroesophageal ALSA		5	28	115	0.96	7.6	-1.82	8.3	-2.16	0.92
**20**	F			5	13	97	0.59	7.2	-0.59	7.6	-1.1	0.95
**21**	F			5	18	108	0.73	7	-1.54	9	-0.89	0.78
**22**	M			7	25.5	118	0.92	6.2	-2.94	7.2	-2.93	0.86
**23**	M			8	43	142	1.31	7.6	-2.38	10	-1.62	0.76
**24**	F			8	22.6	115	0.85	6.7	-2.27	10	-0.72	0.67
**25**	F	Retroesophageal ARSA		9	31	132	1.07	12	0.67	12	-0.15	1.00
**26**	M	RAA		9	30	128	1.01	6.8	-2.61	8.2	-2.36	0.83
**27**	M			9	35	138	1.16	7.9	-1.98	11	-0.83	0.72
**28**	F			9	46	148	1.38	9.9	-0.87	13	-0.12	0.76
**29**	F	Retroesophageal ARSA		10	35	138	1.16	7.9	-1.98	9.1	-1.99	0.86
**30**	M			10	34	134	1.13	9.4	-0.89	11	-0.78	0.85
**31**	M			11	41	145.5	1.29	7.5	-2.44	8.2	-2.8	0.91
**32**	M			11	39	138	1.23	8.8	-1.42	10	-1.51	0.88
**33**	F	RAA		12	45	149	1.37	7	-2.93	8.8	-2.47	0.80
**34**	M			12	34	144	1.16	7.5	-2.29	9	-2.05	0.83
**35**	M	RAA, ALSA	CPAs	12	45	150	1.37	10	-0.8	14	0.35	0.71
**36**	F			13	16	100	0.67	6.1	-2.04	6.5	-2.53	0.94
**37**	M		CPAs	13	57	154	1.5	13	0.73	15	0.47	0.87
**38**	M	RAA, ALSA		14	54	163	1.56	8	-2.32	8.9	-2.76	0.90
**39**	M		CPAs	15	55	170	1.6	8.3	-2.15	12.3	-0.9	0.67
**40**	M			15	51	170	1.54	8.9	-1.66	9.6	-2.25	0.93
**41**	M			16	50	166	1.51	8.3	-2.04	12	-0.83	0.69
**42**	F			18	54	161	1.55	7	-3.11	10	-2.04	0.70
**43**	F			19	63	166	1.71	10.61	-0.85	14.34	-0.32	0.74
**44**	F			19	66	167	1.76	11	-0.71	13.6	-0.81	0.81
**45**	M			19	59	152	1.59	9	-1.65	11	-1.55	0.82
**46**	M			19	70	160	1.78	9.6	-1.58	16	0.07	0.60
**47**	M	RAA, ALSA	CPAs	20	61	176	1.72	7	-3.34	13	-0.93	0.54
**48**	M			20	65	165	1.73	7	-3.37	8	-3.95	0.88
**49**	M		CPAs	21	62	166.5	1.7	12	N.A.	13	N.A.	0.92
**50**	F	RAA, ARSA		21	73	156	1.8	10	N.A.	14	N.A.	0.71
**51**	M			22	61.5	174	1.72	10	N.A.	13	N.A.	0.77
**52**	M			22	69	162	1.78	13.4	N.A.	12.7	N.A.	1.06
**53**	M		CPAs	23	59.5	163	1.65	12.8	N.A.	13.2	N.A.	0.97
**54**	F		CPAs	34	71	165	1.82	7	N.A.	13	N.A.	0.54
**55**	F			34	74	167	1.87	7	N.A.	12	N.A.	0.58
**56**	F			37	91	162	2.06	12	N.A.	16	N.A.	0.56
**57**	M			40	87	172	2.06	8	N.A.	13	N.A.	0.62
**58**	M			45	122	176	2.5	11.4	N.A.	17	N.A.	0.67

Abbreviations: Ao: aortic–PAs: pulmonary arteries; CPAs: crossed pulmonary arteries; DAA: double aortic arch; RAA: right aortic arch; ARSA: aberrant right subclavian artery; ALSA: aberrant left subclavian artery; ret: retroesophageal.

**Table 2:** Echocardiographic data of control patients.

Case	Sex	Ao Arch Anomalies	PAs Anomalies	Age	Weight (kg)	High (cm)	BSA	LPA d	z-score LPA	RPA d	z-score RPA	LPA/RPA
**1**	M			0	9	74	0.44	6.4	0.06	7.3	0.14	0.88
**2**	M			1	12	82	0.53	6	-1.26	6.2	-1.89	0.97
**3**	F			1	12.5	87	0.55	5.9	-1.54	6	-2.28	0.98
**4**	F			1	5.7	70	0.33	9	2.88	8	1.58	1.12
**5**	F			2	10.7	47	0.4	8	1.52	8.6	1.29	0.93
**6**	M			2	15	110	0.67	7.2	-1.06	7.3	-1.84	0.99
**7**	M			2	11.2	90	0.53	6	-1.22	6.8	-1.28	0.88
**8**	M			3	15.5	95	0.64	7.5	-0.67	7.5	-1.53	1
**9**	F			3	15.5	102	0.66	7.2	-1.01	7.5	-1.63	0.96
**10**	F			3	14	90	0.6	6.7	-1.06	7.2	-1.47	0.93
**11**	M			4	21	110	0.8	6.4	-2.37	6.8	-2.9	0.94
**12**	M			4	19	106	0.75	9.6	0.28	9.8	-0.45	0.98
**13**	F			4	15	98	0.64	7.6	-0.57	7.8	-1.26	0.97
**14**	F			4	17	108	0.71	8.1	-0.56	8.6	-1.06	0.94
**15**	M			4	14	103	0.63	6.2	-1.72	6.6	-2.21	0.94
**16**	M			4	23	100	0.81	10.1	0.32	12.2	0.62	0.83
**17**	M			3	18	104	0.72	7.9	-0.77	8.6	-1.12	0.92
**18**	F			5	22	123	0.86	7.6	-1.55	7.5	-2.51	1.01
**19**	M			5	29	126	1.01	9.7	-0.49	11.9	-0.09	0.81
**20**	F			5	26	124	0.95	7.4	-1.95	7.7	-2.59	0.96
**21**	F			5	20	117	0.8	8.5	-0.67	7.5	-2.3	1.13
**22**	M			7	27	121	0.96	8.5	-1.1	10	-0.79	0.85
**23**	M			8	27	138	1.01	8.4	-1.4	8.5	-2.2	0.99
**24**	F			8	25.5	129	0.95	8.4	-1.21	7.9	-2.45	1.06
**25**	F			9	32	128	1.07	7.4	-2.23	7.5	-3.02	0.99
**26**	M			9	40	139	1.25	11	-0.11	11	-0.96	1
**27**	M			9	30.8	138	1.08	8.5	-1.5	9.3	-1.7	0.91
**28**	F			8	35	142	1.17	10.4	-0.35	13.2	0.26	0.79
**29**	F			10	45	150	1.37	9.9	-0.86	9.9	-1.76	1
**30**	M			11	66	162	1.74	12	-0.15	13.4	-0.81	0.89
**31**	M			11	42	158	1.35	10	-0.78	11	-1.09	0.91
**32**	M			11	42	140	1.28	12	0.37	13	0.01	0.92
**33**	F			12	36	148	1.21	10	-0.63	11	-0.9	0.91
**34**	M			12	41	150	1.3	11	-0.17	10	-1.61	1.1
**35**	M			12	40	148	1.28	7.2	-2.68	7.4	-3.41	0.97
**36**	F			13	72	168	1.84	13	0.09	12.8	-1.6	1.02
**37**	M			14	48	164	1.47	13	0.68	10	-1.86	1.3
**38**	M			14	59	170	1.67	9.5	-1.43	9	-2.98	1.05
**39**	M			16	70	163	1.8	10	-1.6	12	-1.3	0.83
**40**	M			16	60	175	1.7	9.3	-1.61	9.3	-2.9	1
**41**	F			18	80	160	1.92	12	1.1	11	-1.9	1.09
**42**	F			20	55	165	1.58	12.12	0.08	13.58	-0.24	0.89
**43**	M			20	68	168	1.79	8	-2.68	8.6	-3.74	0.93
**44**	M			20	67	172	1.79	10	-1.36	10	-2.83	1
**45**	M			21	82	190	2.08	11	N.A.	9	N.A.	1.2
**46**	F			22	85	165	2.00	7	N.A.	7.1	N.A.	0.98
**47**	M			22	76	177	1.94	9.1	N.A.	10	N.A.	0.91
**48**	M			21	63	170	1.73	9.8	N.A.	11	N.A.	0.89
**49**	M			23	87	170	2.05	14	N.A.	15	N.A.	0.93
**50**	F			33	58	170	1.65	12.69	N.A.	13.36	N.A.	0.95
**51**	F			33	61	162	1.66	13	N.A.	14	N.A.	0.93
**52**	F			38	78	170	1.94	13	N.A.	13.8	N.A.	0.94
**53**	M			40	107	170	2.29	17	N.A.	16	N.A.	1.06
**54**	M			45	90	178	2.13	17.1	N.A.	19	N.A.	0.9

Abbreviations: Ao: aortic—PAs: pulmonary arteries—RAA: right aortic arch–ARSA: aberrant right subclavian artery–DAA: double aortic arch–ALSA: aberrant left subclavian artery.

### *Tbx1* haploinsufficiency is associated with smaller LPAs in mice

**Fig 1:**
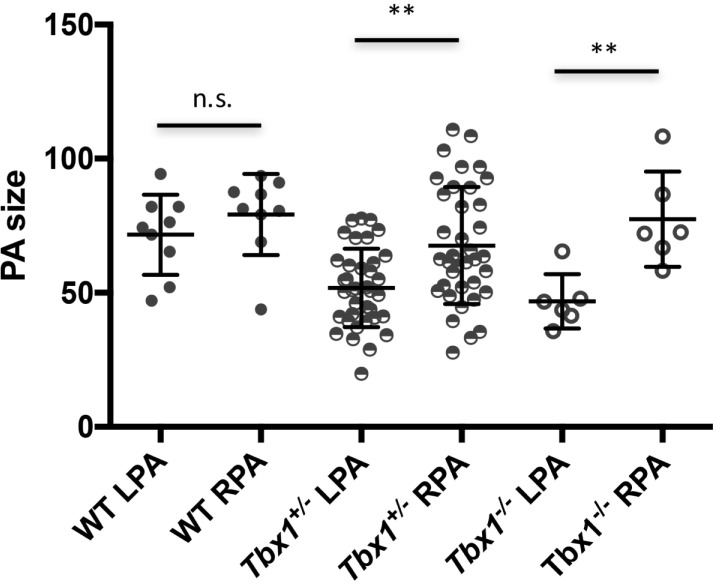
Pulmonary artery size in mouse fetuses. Distribution of pulmonary arteries measurements in WT, *Tbx1*^+/-^, and *Tbx1*^-/-^ fetuses at E18.5. n.s.: not significant; **: P value < 0.005, Mann-Whitney test. The data source used to generate this graph is in the Supporting Information Table 1.

*TBX1* is the candidate gene for many of the clinical and developmental features of 22q11.2DS patients including aortic arch anomalies and intracardiac anomalies. However, to our knowledge, anomalies of PAs in mouse mutants have not been reported to date. To understand whether loss of *Tbx1* may be a candidate also for the observed size asymmetry of the PAs, we measured them in *Tbx1*^+/+^, *Tbx1*^+/-^, and *Tbx1*^-/-^ E18.5 fetuses in a homogeneous congenic background C57Bl6/N. Results are plotted in Fig 1. In WT (*Tbx1*^+/+^) fetuses, we found no significant difference (Mann-Whitney test) in the diameters of LPAs and RPAs (ratio LPA/RPA = 0.92. n = 8). However, in *Tbx1*^+/-^ fetuses, the LPAs were significantly smaller than the RPAs (P = 0.0016, ratio LPA/RPA = 0.79, n = 37). Similarly, the *Tbx1*^-/-^ fetuses also had significantly different PAs (P = 0.004, ratio LPA/RPA = 0.63, n = 6). All WT fetuses had normal arch and epiaortic vessels. Of the 37 heterozygous animals analyzed, 14 had aberrant origin of the right subclavian artery (37.8%) of which, 2 had high aortic arch, 3 interrupted aortic arch type B, and 1 right aortic arch. All 6 *Tbx1*^-/-^ fetuses had truncus arteriosus, as previously described [15]. In all *Tbx1*^-/-^ fetuses the pulmonary arteries rose separately from the posterior wall of the arterial trunk proximal to the branches of aortic arch (Truncus Arteriosus—type II of Collett and Edwards or type A2 of Van Praagh).

### Tbx1-expressing cells contribute to structural components of the pulmonary arteries

**Fig 2:**
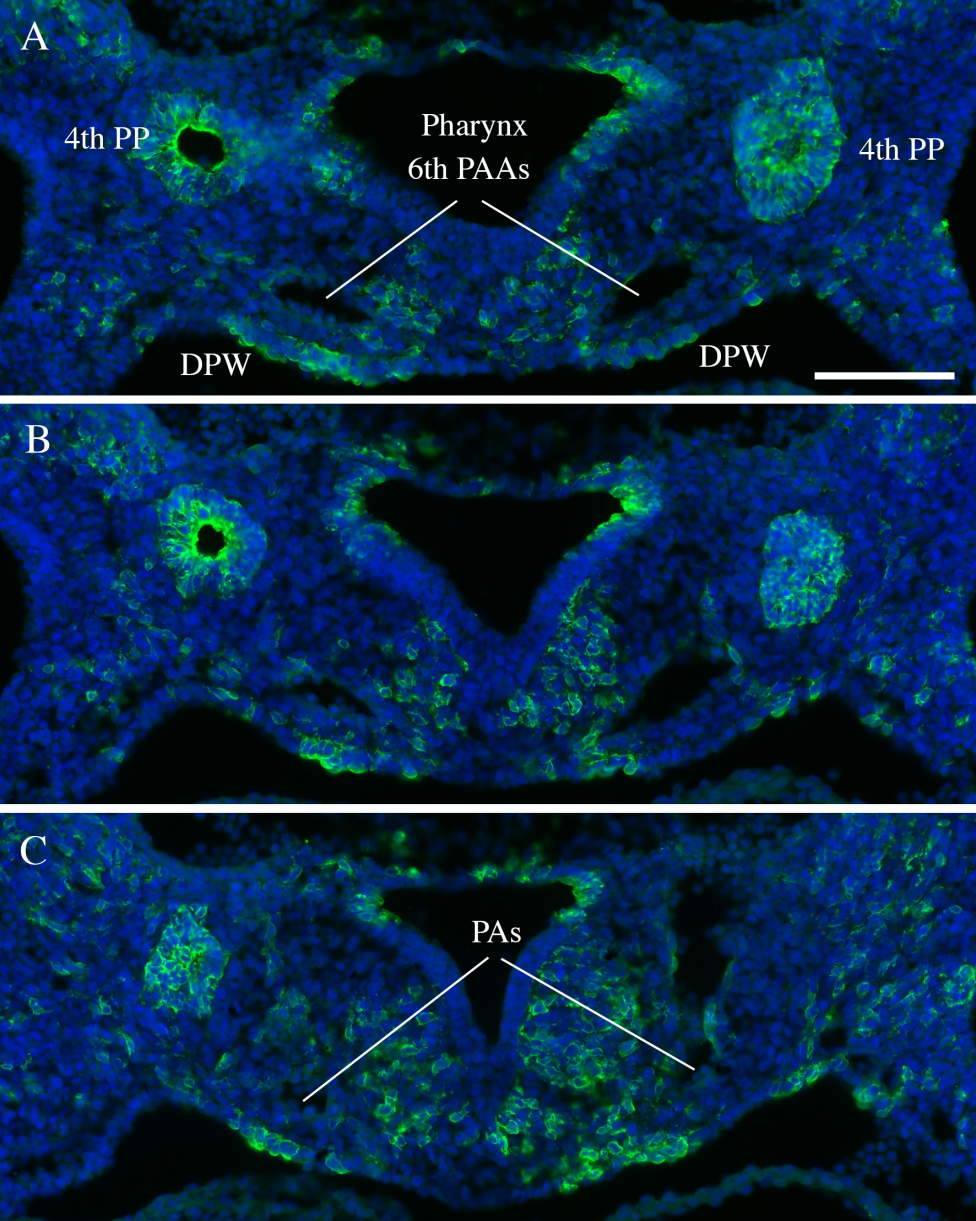
*Tbx1* expression in mouse embryos. Transverse section of a E10.5 *Tbx1*^*cre*/+^; *Rosa*^*mT*/mG^ embryos immunostained with an anti GFP antibody (green). GFP positivity indicate cells that have expressed Cre recombinase. A, B, and C refer to 3 adjacent sections (cranial -> caudal) that span the junction between the 6th pharyngeal arch arteries (PAAs) and the putative pulmonary arteries (PAs). PP: pharyngeal pouches; DPW: dorsal pericardial wall. Scale bar is 100 micrometers.

**Fig 3:**
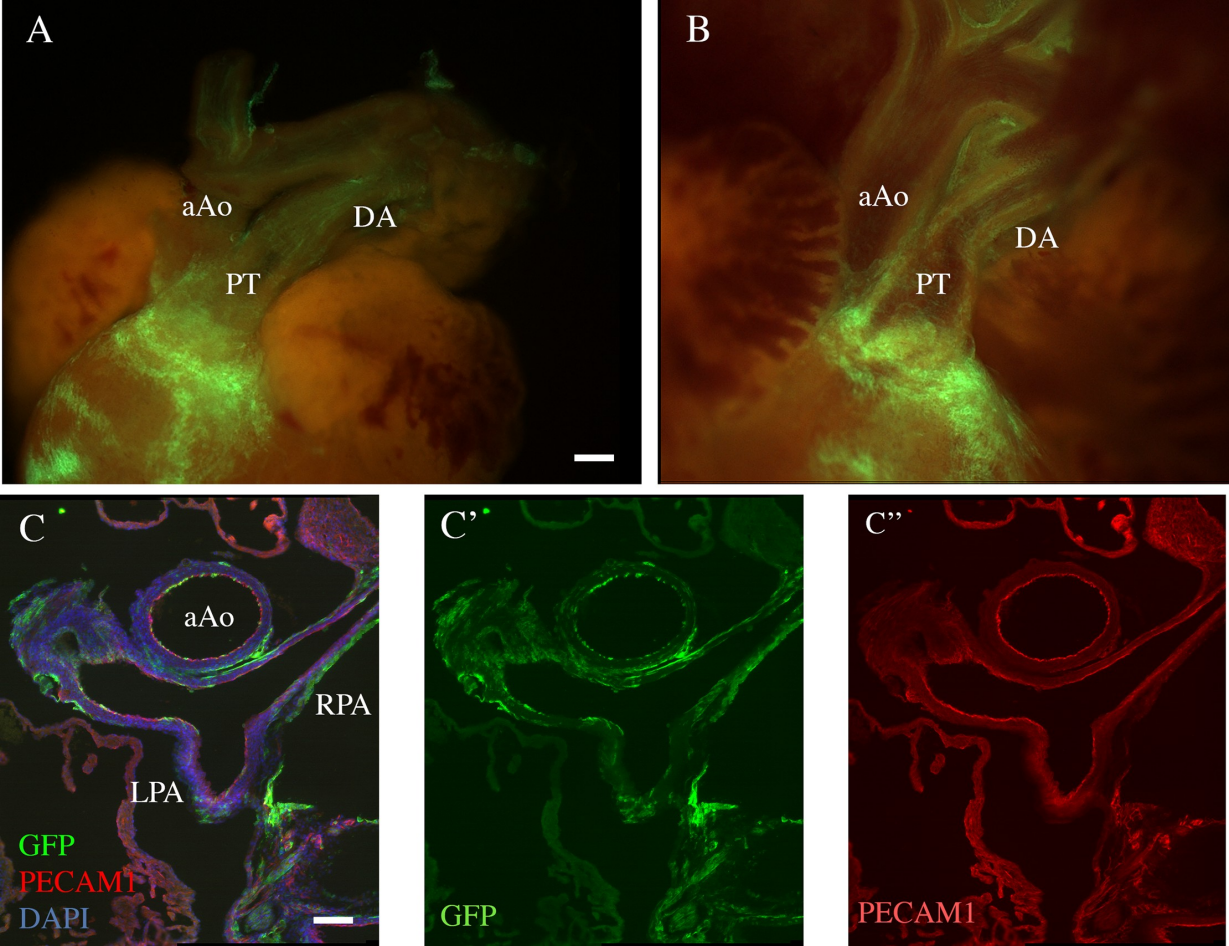
Distribution of Tbx1-expressing cells and their descendants in mouse fetuses. A.B: Whole mount fluorescent photographs of the outflow region of a E18.5 *Tbx1*^*cre*/+^; *Rosa*^*mT*/mG^ fetus. A: external appearance. B: internal optical plane. Note the heavy contribution of GFP+ cells to the pulmonary myocardium, pulmonary trunk (PT) and pulmonary valves, but superficial contribution (endothelial and adventitial) to other great vessels, including the ductus arteriosus (DA). which appears to have a more dense endothelial contribution. aAo: ascending aorta. C: immunofluorescence of a transverse section of a E18.5 *Tbx1*^*cre*/+^; *Rosa*^*mT*/mG^. Anti GFP staining is shown in green, anti PECAM1 staining (endothelial-specific) is shown in red. DAPI staining (cell nuclei) is shown in blu. C'-C'': green and red channels are shown separately. aAo: ascending aorta; PT: pulmonary trunk and pulmonary leaflets; LPA, RPA: left and right pulmonary arteries. Scale bar is 50 micrometers in A and B, 100 micrometers in C-C".

To provide insights as to how *Tbx1* may affect the development of the PAs, we looked into the expression of the gene. To do this. we used genetic marking of *Tbx1*-expressing cells and their descendants in *Tbx1*^*cre*/+^; *Rosa*^*mT*/mG^ embryos in which these cells are marked by membrane-bound green fluorescent protein (GFP). At E10.5, the PAs connect the aortic sac (through the proximal end of the 6th pharyngeal arch arteries) to the lung buds. GFP+ cells were observed in the mesoderm adjacent to the arteries, in the adjacent dorsal pericardial wall, and in the inner, endothelial layer of the arteries (Fig 2). In the mature PAs at E18.5, the distribution of GFP+ cells were observed in the endothelial layer and in the outer mesenchymal tissue adjacent to the arteries (Fig 3). We did not observe contribution of GFP+ cells in the smooth muscle layer of the arteries, in contrast to the pulmonary trunk.

## Discussion

The junction between LPA and DA is a crucial segment for the cardiovascular development and it is frequently affected in patients with conotruncal anomalies [29–32]. Also in healthy people a smaller diameters of the LPA was reported in comparison with the RPA. Moreover, malformations of the pulmonary arteries, in particular of the left, including stenosis, diffuse hypoplasia, discontinuity or crossing, are not unusual in children with 22q11.2DS with or without conotruncal defects [33, 34, 3, 35, 36, 25, 24, 37]. The detailed morphogenesis of the pulmonary arteries is not definitively ascertained. However, recent studies on mouse and human embryos contribute to better clarify this difficult topic [38, 39]. According to recent data, while on the right side the VI pharyngeal arch artery disappears, on the left side it is formed by a ventral bud from the aortic sac and by a dorsal bud from the dorsal aorta. This ventral bud, with the contribution from the post-branchial pulmonary plexus, forms the LPA. The dorsal bud on the left side of the VI aortic arch forms the ductus arteriosus (DA), which is in continuity with the LPA. Our echocardiographic studies show that even in the absence of conotruncal defects, patients with 22q11.2DS have a smaller LPA compared to healthy subjects.

Our data demonstrate that the LPA is smaller than the RPA in *Tbx1*^+/-^ fetuses but not in WT fetuses, indicating that *Tbx1* haploinsufficiency affects significantly the LPA size. Expression data indicate that structural components of the PAs (endothelium and adventitia) derive from *Tbx1*-expressing cells. This is also true for the DA, thus suggesting that Tbx1 is involved in the development or growth of this cardiovascular segment. Mouse data are in agreement with echocardiographic measurements on patients with 22q11.2DS.

It is of interest to note that in mice *Tbx1* haploinsufficiency affects the IV but not the VI aortic arch artery development [19, 40], while in *Tbx1*^-/-^ embryos the VI does not develop [19]. The phenotype that we have described here suggest that a) the absence of the VI aortic arches does not have a dramatic impact on PAs development, and b) the reduced size of the LPA is probably not secondary to abnormalities of the VI aortic arch. The finding that *Tbx1*-expressing cells contribute to structural component of the PAs provides a support for a direct, though limited role of *Tbx1* in determining the size of the PAs.

In the past, stenosis, diffuse hypoplasia or atresia of the proximal LPA was mainly ascribed to the extension of the ductal tissue into the LPA lumen. This pathogenetic mechanism known as “coarctation of the LPA” maintains its validity. However, our data suggest that molecular causes may influence the morphogenesis of this peculiar cardiovascular region, in particular the effect of *Tbx1* in this region may influence the morphology and dimensions of the LPA and its loss of function may cause some of its specific defects.

The reduced dimensions of LPA observed in our patients could be considered a subclinical sign associated with 22q11.2DS. We suggest that in subjects with 22q11.2DS the junction between the DA and the LPA may be at risk of hypoplasia or additional anomalies and deserves specific diagnostic investigation, also in patients with conotruncal defects.

## Supporting information

S1 Table Measurements used to generate the graph shown in Fig 1. (XLSX)

## References

[1] Momma K. Kondo C. Matsuoka R. Takao A. Cardiac anomalies associated with a chromosome 22q11 deletion in patients with conotruncal anomaly face syndrome. Am J Cardiol. 1996 9 1;78(5):591–4. 8806353 10.1016/s0002-9149(96)00374-8

[2] Goldmuntz E. Clark BJ. Mitchell LE. Jawad AF. Cuneo BF. Reed L. et al Frequency of 22q11 deletions in patients with conotruncal defects. J Am Coll Cardiol. 1998 8;32(2):492–8. 9708481 10.1016/s0735-1097(98)00259-9

[3] Marino B. Digilio MC. Toscano A. Giannotti A. Dallapiccola B. Congenital heart defects in patients with DiGeorge/velocardiofacial syndrome and del22q11. Genet Couns. 1999;10(1):25–33. 10191426

[4] Momma K. Kondo C. Ando M. Matsuoka R. Takao A. Tetralogy of Fallot associated with chromosome 22q11 deletion. Am J Cardiol. 1995 9 15;76(8):618–21. 10.1016/s0002-9149(99)80170-2 7677092

[5] Marino B. Digilio MC. Grazioli S. Formigari R. Mingarelli R. Giannotti A. et al Associated cardiac anomalies in isolated and syndromic patients with tetralogy of Fallot. Am J Cardiol. 1996 3 1;77(7):505–8. 10.1016/s0002-9149(97)89345-9 8629592

[6] Momma K. Ando M. Matsuoka R. Truncus arteriosus communis associated with chromosome 22q11 deletion. J Am Coll Cardiol. 1997 10;30(4):1067–71. 9316541 10.1016/s0735-1097(97)00240-4

[7] Marino B. Digilio MC. Toscano A. Common arterial trunk. DiGeorge syndrome and microdeletion 22q11. Prog Pediatr Cardiol. 2002 6;15(1):9–17.

[8] Lewin MB. Lindsay EA. Jurecic V. Goytia V. Towbin JA. Baldini A. A genetic etiology for interruption of the aortic arch type B. Am J Cardiol. 1997 8 15;80(4):493–7. 9285664 10.1016/s0002-9149(97)00401-3

[9] Marino B. Digilio MC. Persiani M. Di Donato R. Toscano A. Giannotti A. et al Deletion 22q11 in patients with interrupted aortic arch. Am J Cardiol. 1999 8 1;84(3):360–1. A9. 10496456 10.1016/s0002-9149(99)00297-0

[10] Momma K. Matsuoka R. Takao A. Aortic Arch Anomalies Associated with Chromosome 22q11 Deletion (CATCH 22). Pediatr Cardiol. 1999 3 11;20(2):97–102. 10.1007/s002469900414 9986884

[11] McElhinney DB. Clark BJ. Weinberg PM. Kenton ML. McDonald-McGinn D. Driscoll DA. et al Association of chromosome 22q11 deletion with isolated anomalies of aortic arch laterality and branching. J Am Coll Cardiol. 2001 6 15;37(8):2114–9. 11419896 10.1016/s0735-1097(01)01286-4

[12] Toscano A. Anaclerio S. Digilio MC. Giannotti A. Fariello G. Dallapiccola B. et al Ventricular septal defect and deletion of chromosome 22q11: anatomical types and aortic arch anomalies. Eur J Pediatr. 2002 2;161(2):116–7. 11954747 10.1007/s00431-001-0877-5

[13] Edelmann L. Pandita RK. Morrow BE. Low-copy repeats mediate the common 3-Mb deletion in patients with velo-cardio-facial syndrome. Am J Hum Genet. 1999 4;64(4):1076–86. 10090893 10.1086/302343 PMC1377832

[14] Lindsay EA. Botta A. Jurecic V. Carattini-Rivera S. Cheah YC. Rosenblatt HM. et al Congenital heart disease in mice deficient for the DiGeorge syndrome region. Nature. 1999 9 23;401(6751):379–83. 10.1038/43900 10517636

[15] Lindsay EA. Vitelli F. Su H. Morishima M. Huynh T. Pramparo T. et al Tbx1 haploinsufficieny in the DiGeorge syndrome region causes aortic arch defects in mice. Nature. 2001 3 1;410(6824):97–101. 10.1038/35065105 11242049

[16] Jerome LA. Papaioannou VE. DiGeorge syndrome phenotype in mice mutant for the T-box gene. Tbx1. Nat Genet. 2001 3;27(3):286–91. 10.1038/85845 11242110

[17] Merscher S. Funke B. Epstein JA. Heyer J. Puech A. Lu MM. et al TBX1 is responsible for cardiovascular defects in velo-cardio-facial/DiGeorge syndrome. Cell. 2001 2 23;104(4):619–29. 11239417 10.1016/s0092-8674(01)00247-1

[18] Yagi H. Furutani Y. Hamada H. Sasaki T. Asakawa S. Minoshima S. et al Role of TBX1 in human del22q11.2 syndrome. Lancet (London. England). 2003 10 25;362(9393):1366–73. 10.1016/s0140-6736(03)14632-6 14585638

[19] Vitelli F. Morishima M. Taddei I. Lindsay EA. Baldini A. Tbx1 mutation causes multiple cardiovascular defects and disrupts neural crest and cranial nerve migratory pathways. Hum Mol Genet. 2002 4 15;11(8):915–22. 11971873 10.1093/hmg/11.8.915

[20] Kelly RG. Papaioannou VE. Visualization of outflow tract development in the absence of Tbx1 using an FgF10 enhancer trap transgene. Dev Dyn. 2007 3;236(3):821–8. 10.1002/dvdy.21063 17238155

[21] Rana MS. Theveniau-Ruissy M. De Bono C. Mesbah K. Francou A. Rammah M. et al Tbx1 Coordinates Addition of Posterior Second Heart Field Progenitor Cells to the Arterial and Venous Poles of the Heart. Circ Res. 2014 9;115(9):790–9. 10.1161/CIRCRESAHA.115.305020 25190705

[22] Xu H. Morishima M. Wylie JN. Schwartz RJ. Bruneau BG. Lindsay EA. et al Tbx1 has a dual role in the morphogenesis of the cardiac outflow tract. Development. 2004 7;131(13):3217–27. 10.1242/dev.01174 15175244

[23] Aggarwal V. Imamura M. Acuna C. Cabrera AG. Chromosome 22q11 deletion in a patient with pulmonary atresia. intact ventricular septum. and confluent branch pulmonary arteries. Cardiol Young. 2017 12 13;1–4. 10.1017/S104795111700227X 29233233

[24] Maldjian P. Sanders A. 22q11 Deletion Syndrome with Vascular Anomalies. J Clin Imaging Sci. 2018;8(1):1. 29441224 10.4103/jcis.JCIS_66_17 PMC5801571

[25] Romano R. Cirillo E. Giardino G. Gallo V. Mollica C. Pignata C. A Bronchovascular Anomaly in a Patient With 22q11.2 Deletion Syndrome. J Investig Allergol Clin Immunol. 2016 12 19;26(6):390–2. 10.18176/jiaci.0107 27996952

[26] Babaoğlu K. Altun G. Binnetoğlu K. Dönmez M. Kayabey Ö. Anık Y. Crossed Pulmonary Arteries: A Report on 20 Cases With an Emphasis on the Clinical Features and the Genetic and Cardiac Abnormalities. Pediatr Cardiol. 2013 12 10;34(8):1785–90. 10.1007/s00246-013-0714-4 23660850

[27] Huynh T. Chen L. Terrell P. Baldini A. A fate map of Tbx1 expressing cells reveals heterogeneity in the second cardiac field. genesis. 2007 7;45(7):470–5. 10.1002/dvg.20317 17610275

[28] Muzumdar MD. Tasic B. Miyamichi K. Li L. Luo L. A global double-fluorescent Cre reporter mouse. Genes (New York. NY 2000). 2007 9;45(9):593–605. 10.1002/dvg.20335 17868096

[29] Momma K. Takao A. Ando M. Nakazawa M. Satomi G. Imai Y. et al Juxtaductal left pulmonary artery obstruction in pulmonary atresia. Br Heart J. 1986 1;55(1):39–44. 3947479 10.1136/hrt.55.1.39 PMC1232066

[30] Elzenga NJ. Gittenberger-de Groot AC. The ductus arteriosus and stenoses of the pulmonary arteries in pulmonary atresia. Int J Cardiol. 1986 5;11(2):195–208. 3710625 10.1016/0167-5273(86)90179-8

[31] Elzenga NJ. von Suylen RJ. Frohn-Mulder I. Essed CE. Bos E. Quaegebeur JM. Juxtaductal pulmonary artery coarctation. An underestimated cause of branch pulmonary artery stenosis in patients with pulmonary atresia or stenosis and a ventricular septal defect. J Thorac Cardiovasc Surg. 1990 9;100(3):416–24. 2391977

[32] Presbitero P. Bull C. Haworth SG. de Leval MR. Absent or occult pulmonary artery. Br Heart J. 1984 8;52(2):178–85. 6743435 10.1136/hrt.52.2.178 PMC481607

[33] Kutsche LM. Van Mierop LH. Anomalous origin of a pulmonary artery from the ascending aorta: associated anomalies and pathogenesis. Am J Cardiol. 1988 4 1;61(10):850–6. 3354450 10.1016/0002-9149(88)91078-8

[34] Momma K. Kondo C. Matsuoka R. Tetralogy of Fallot with pulmonary atresia associated with chromosome 22q11 deletion. J Am Coll Cardiol. 1996 1;27(1):198–202. 10.1016/0735-1097(95)00415-7 8522695

[35] Dodo H. Alejos JC. Perloff JK. Laks H. Drinkwater DC. Williams RG. Anomalous origin of the left main pulmonary artery from the ascending aorta associated with DiGeorge syndrome. Am J Cardiol. 1995 6 15;75(17):1294–5. 7778565 10.1016/s0002-9149(99)80788-7

[36] Sett SS. Sandor GG. Mawson JB. Interrupted right aortic arch and origin of the left pulmonary artery from the aorta in DiGeorge syndrome. Cardiol Young. 2001 11;11(6):676–9. 11813925 10.1017/s104795110100110x

[37] Pettersen MD. Du W. Skeens ME. Humes RA. Regression Equations for Calculation of Z Scores of Cardiac Structures in a Large Cohort of Healthy Infants. Children. and Adolescents: An Echocardiographic Study. J Am Soc Echocardiogr. 2008 8;21(8):922–34. 10.1016/j.echo.2008.02.006 18406572

[38] Hiruma T. Nakajima Y. Nakamura H. Development of pharyngeal arch arteries in early mouse embryo. J Anat. 2002 7;201(1):15–29. 10.1046/j.1469-7580.2002.00071.x 12171473 PMC1570898

[39] Rana MS. Sizarov A. Christoffels VM. Moorman AFM. Development of the human aortic arch system captured in an interactive three-dimensional reference model. Am J Med Genet A. 2014 6;164A(6):1372–83. 10.1002/ajmg.a.35881 23613216

[40] Zhang Z. Cerrato F. Xu H. Vitelli F. Morishima M. Vincentz J. et al Tbx1 expression in pharyngeal epithelia is necessary for pharyngeal arch artery development. Development. 2005 12;132(23):5307–15. 10.1242/dev.02086 16284121

